# Controlled Delivery of Gentamicin Using Poly(3-hydroxybutyrate) Microspheres

**DOI:** 10.3390/ijms12074294

**Published:** 2011-07-04

**Authors:** Lydia Francis, Decheng Meng, Jonathan Knowles, Tajalli Keshavarz, Aldo R. Boccaccini, Ipsita Roy

**Affiliations:** 1 Department of Applied and Molecular Biosciences, School of Life Sciences, University of Westminster, 115 New Cavendish Street, London W1W 6UW, UK; E-Mails: lydiafrancis12@gmail.com (L.F.); keshavt@wmin.ac.uk (T.K.); 2 Department of Materials, Imperial College London Prince Consort Rd, London SW7 2AZ, UK; E-Mail: d.meng07@imperial.ac.uk; 3 Division of Biomaterials and Tissue Engineering, UCL Eastman Dental Institute, University College London 256 Gray’s Inn Road, London WC1X 8LD, UK; E-Mail: j.knowles@ucl.ac.uk; 4 WCU Research Centre of Nanobiomedical Science, Dankook University, San#29, Anseo-dong, Dongnam-gu, Cheonan-si, Chungnam, 330-714, Korea; 5 Department of Materials Science and Engineering, Institute of Biomaterials, University of Erlangen-Nuremberg, 91058 Erlangen, Germany

**Keywords:** Poly(3-hydroxybutyrate), microspheres, controlled drug delivery, gentamicin

## Abstract

Poly(3-hydroxybutyrate), P(3HB), produced from *Bacillus cereus* SPV using a simple glucose feeding strategy was used to fabricate P(3HB) microspheres using a solid-in-oil-water (s/o/w) technique. For this study, several parameters such as polymer concentration, surfactant and stirring rates were varied in order to determine their effect on microsphere characteristics. The average size of the microspheres was in the range of 2 μm to 1.54 μm with specific surface areas varying between 9.60 m^2^/g and 6.05 m^2^/g. Low stirring speed of 300 rpm produced slightly larger microspheres when compared to the smaller microspheres produced when the stirring velocity was increased to 800 rpm. The surface morphology of the microspheres after solvent evaporation appeared smooth when observed under SEM. Gentamicin was encapsulated within these P(3HB) microspheres and the release kinetics from the microspheres exhibiting the highest encapsulation efficiency, which was 48%, was investigated. The *in vitro* release of gentamicin was bimodal, an initial burst release was observed followed by a diffusion mediated sustained release. Biodegradable P(3HB) microspheres developed in this research has shown high potential to be used in various biomedical applications.

## 1. Introduction

Polymeric drug delivery systems are designed to deliver drugs to the local site of action for extended periods of time, so that the therapeutic levels of drugs with short *in vivo* half-lives can be maintained [1]. In addition, different forms of drug delivery devices are used to reduce the fluctuations in plasma drug levels, so a slower and controlled drug release rate can be achieved, which can then provide an effective pharmacological response [1,2]. Microencapsulation of a drug within a polymeric device, e.g., microspheres is considered as one of the most common methods of drug delivery [3]. Drug encapsulated microspheres require less frequent drug administration when compared to the conventional dosage forms. Also, drugs encapsulated within microspheres are kept separate from other microspheres; as a result multiple drug administration in a single injection can be achieved, which would have not been possible otherwise, owing to drug compatibility issues [1]. Apart from being orally administered, microspheres can also be administered through a number of parenteral pathways such as intraocular, intravenous, intra-arterial, intraspinal and intraosseous [3]. Often the site of administration influences the performance of drug delivery due to differences in the local tissue environment such as pH and enzyme activity [3].

The solvent evaporation method is the most frequently used technique to prepare polymeric microspheres for drug delivery. The characteristics of the microspheres produced are largely dependent on the type of polymer (hydrophobic/hydrophilic) and drug used. Other processing parameters such as solvent type and concentration of the emulsifier, drug/polymer ratio and stirring rate are also known to affect the physicochemical properties of the microspheres [4]. For example, surface morphology and porosity of hydrophobic microspheres are known to be particularly affected by the processing parameters due to which the drug release rate from microspheres is also affected. However, the encapsulation efficiency of drugs within hydrophobic microspheres is largely dependent on the type of microencapsulation procedure and on the solubility of the drug. For example, in the single emulsion method (o/w), a predetermined quantity of the polymer dissolved in the solvent is added to deionized water (inner aqueous phase, w_1_) containing polyvinyl alcohol PVA, which is used as a surfactant for the production of microspheres. The solvent is removed from the inner aqueous phase and evaporated through the emulsion-air interface. Polymer precipitation is accelerated leading to the formation of the final microsphere, once the solvent evaporation is initiated. However, the encapsulation efficiency of highly hydrophilic drugs is affected by the fast shrinkage of the microspheres. As a result, the encapsulated drug is drained out of the microspheres during solvent removal. Therefore the o/w method is more suitable for the entrapment of less hydrophilic drugs as they accumulate towards the microsphere surface due to their hydrophobic nature. In the double emulsion method (w_1_/o/w_2_) the initial emulsion phase (w_1_) is added to a second aqueous phase containing PVA (w_2_). During solvent evaporation, small microdroplets formed within the microspheres coalesce forming a honeycomb structure. During solvent evaporation, the precipitating polymer wall also forms holes through which the entrapped drug is partly removed. This honeycomb structure is considered most suitable for efficient entrapment of hydrophilic drugs [5].

The double emulsion method has been used by several researchers to entrap highly hydrophilic drugs such as gentamicin within biodegradable hydrophobic microspheres [6,7]. Gentamicin, a highly water soluble drug, is an aminoglycoside that has been used against a wide range of Gram-positive and Gram-negative bacteria [7]. For example, gentamicin has been used for the treatment of osteomyelitis, an inflammatory bone disease [7]. Osteomyelitis is the microbial infection of the bone medullary cavity, cortex and periosteum that is known to occur during post-operative sepsis after an orthopaedic procedure. However, for the treatment of osteomyelitis, a prolonged systemic antibiotic treatment such as the use of gentamicin, either oral or parenteral, for a period of 4–6 weeks, is known to cause systemic toxicity and patient discomfort. Therefore, localised drug delivery at the infected site, for example injecting drug loaded microspheres, is being proposed. By this method the risk of high dose administration and possibility of building drug resistance could be avoided [8]. In a study conducted by Huang *et al.* [8] PLA microspheres containing gentamicin were developed for the treatment of bone infection. PLA microspheres with an average size of 178 μm were incorporated with gentamicin sulphate for the treatment of osteomyelitis, where 80% of gentamicin sulphate was released within 3 weeks of implantation [8].

Although these biodegradable polymers such as PLA prove to be advantageous since they can circumvent some of the problems faced when long term implants are used, they have a risk of toxicity and immunogenicity due to their acidic by-products. Other properties such as high price, lack of tailorability, presence of chemical catalysts and fast degradation rate has challenged their commercialization [9]. Thus the need for polyhydroxyalkanoates has arisen due to their tailorable mechanical properties, biocompatibility and biodegradability.

In this paper, P(3HB) microspheres containing gentamicin for the treatment of bone infections such as osteomyelitis were developed. Localised drug delivery at the site of infection using these drug loaded microspheres is being proposed, for example incorporating the microspheres into 3D porous scaffolds [10]. The main advantage of the P(3HB) microspheres is the lack of risk of toxicity and immunogenicity due to acidic by products, which is a disadvantage when PLA is used [11,12]. In this work, the processing conditions that particularly affect the characteristics of the P(3HB) microspheres were investigated so that microspheres with a high gentamicin loading efficiency, controllable porosity and uniform drug distribution could be produced.

## 2. Materials and Methods

### 2.1. Materials

Distilled water and high performance liquid chromatography (HPLC) grade water were used where appropriate. The two types of water were obtained from Elga Pure Lab Options distillation units. All chemicals, buffers and drugs used were purchased from Sigma-Aldrich (Dorset UK). Care was taken to make sure that the chemicals used were of the best grade available (analytical or HPLC where appropriate) for the experiment.

P(3HB) was isolated from the Gram-positive bacteria *Bacillus cereus SPV* which was obtained from the culture collection of University of Westminster, London, UK. All chemicals required for the growth of *Bacillus cereus* SPV and extraction of polymer from the bacterial cells were obtained from Sigma-Aldrich Company Ltd. and VWR Chemicals (England) except for nutrient broth and yeast extract, which was obtained from DIFCO (BD UK Ltd., Oxford, UK). Antifoam (FG-10) was purchased from Dow corning (Edison, NJ, USA) for fermentation study.

### 2.2. P(3HB) Microsphere Production

A solid-in-oil-in-water emulsion technique was used for P(3HB) microsphere preparation. Quantities of 1 or 3 g of P(3HB) were dissolved in 8 mL of chloroform and agitated for 3 min. This mixture was then transferred into the first solid-in-oil emulsion (w/o) of 40 mL of 1% or 0.5% w/v aqueous polyvinyl alcohol (PVA) solution, and stirred at 1000 rpm for 3 min. This solution was then added to a second solid-in-oil-in-water emulsion (w/o/w) of 500 mL of 0.5% or 0.05% w/v aqueous PVA solution, forming the second oil-in-water emulsion. This emulsion was stirred either at 300 rpm or 800 rpm for 4 h to eliminate the chloroform used as solvent and then to form P(3HB) microspheres. The resulting microspheres were isolated by centrifugation at 3680 g for 5 min and then washed with distilled water three times, air dried and stored in a desiccator until further use. Table 1 shows the varying processing conditions (amount of surfactant, polymer concentration and stirring rate) used for the synthesis of P(3HB) microspheres.

### 2.3. Gentamicin Loaded P(3HB) Microsphere Production

Gentamicin-loaded P(3HB) microspheres were produced using similar methods as described in section 2.2. In this case, 2 mg/g of P(3HB) mixed with gentamicin were dissolved in 8 mL of chloroform and agitated for 3 min. This solution was then added to the aqueous first solid-in-oil emulsion of 40 mL of 1% or 0.5% w/v aqueous polyvinyl alcohol (PVA) solution, and stirred at 1000 rpm for 3 min. This solution was then added to a second solid-in-oil-in-water emulsion of 500 mL of 0.5% or 0.05% w/v aqueous PVA solution, forming the second oil-in-water emulsion. This emulsion was stirred either at 300 rpm or 800 rpm for 4 h to eliminate the chloroform used as solvent and to form P(3HB) microspheres. The resulting microspheres were isolated by centrifugation at 3680 g for 5 min and then freeze-dried (Savant Modulyo D Freeze-drier, Thermo Electron Corp, K) and stored at 4 °C until further use.

### 2.4. *In Vitro* Drug Release Studies

The *in vitro* drug release experiments were performed in an incubator at 37 °C for 24 h. 10 mg of the gentamicin-loaded microspheres were immersed in 2 mL of simulated body fluid (SBF) and 1 mL samples were collected at regular intervals, up to a final duration of 24 h. SBF was prepared following the study of Kukobo *et al.* [13]. SBF was used as it has similar ionic concentration as blood plasma. It is therefore important to identify whether or not the gentamicin encapsulated within the microspheres was released in a controlled manner when implanted *in vivo*. Each aliquot was replaced with fresh buffer and the tubes returned to the shaker. At each time point the samples were taken out in triplicates and the results averaged. The drug content was determined by injecting 15 μL of the samples onto a high-performance liquid chromatography (HPLC) column and the gentamicin concentration quantified using the standard curve. The experiments were repeated three times.

### 2.5. Drug Quantification Methods

The encapsulation efficiency (EE%) of the drug-loaded microspheres prepared under different conditions as mentioned in Section 2.2. was determined using Equation 1.

(1) EE%=experimental drug loading/actual drug loading

To determine the EE values, 5 mg of the drug loaded microspheres were dissolved in 1 mL of chloroform, to which 5 mL of water was added after the microspheres were well dissolved. Drugs used in this study, being hydrophilic in nature, separated into the water phase on vortexing. The water phase was then analysed for the drug content using liquid chromatography-mass spectrometry (LC–MS).

#### 2.5.1. Liquid Chromatography Mass Spectrometry

Liquid Chromatography Mass Spectrometry (LC-MS) was used for analysing the drug content from the release buffer. 15 μL samples collected at different time points were injected onto the HPLC column (Dionex HPLC) coupled with an atmospheric pressure (AP)-electrospray ionization (ESI) mass spectrometer (Dionex). Separation was carried out at 50 °C on a reversed-phase C18 60 RP column of dimensions 250 mm × 4 mm [7].

#### 2.5.2. Mobile Phase used for Gentamicin Quantification

The mobile phase used for gentamicin quantification was an isocratic flow of 60% pentafluoropropionic acid (20 mM in ultra-pure water from Fluka Chemicals, Bucks, Switzerland) and 40% methanol. The standard curve was plotted for gentamicin concentrations ranging between 1.0 and 2000 μg mL^−1^ [7].

### 2.6. P(3HB) Microsphere Characterization Techniques

#### 2.6.1. Particle size Analysis

Malvern Mastersizer particle size analyser (Worcestershire, UK) was used to measure the particle size of the microspheres. Microspheres were dispersed well in water and following a background measurement; the suspensions were added drop wise to the analyser until the ideal concentration was reached.

#### 2.6.2. Surface Morphology and Microstructure Characterization

P(3HB) microspheres were observed using a JEOL 5610LV scanning electron microscope (SEM). The samples were placed on 8 mm diameter aluminum stubs using a sticky tag to hold the sample. A gold sputtering device (EMITECH-K550) was used to coat the samples, operating at a pressure of 7 × 10^−2^ bar and deposition current of 20 mA for 2 min; images were taken at various magnifications to analyse the samples.

#### 2.6.3. Porosity

The porosity (ɛ) of the microspheres was measured using the liquid displacement method. Briefly, 5 mL of ethanol was used as the displacement liquid in a measuring cylinder and weighed. The P(3HB) microspheres immersed in ethanol in the cylinder were sonicated in a water bath to assist penetration of ethanol within the pores.

(2) $$V_{p}(\text{the volume of the matrix pores})=(W_{2}-W_{3}-W_{S})/\rho_{e}$$

(3) $$V_{s}(\text{the volume of the matrix polymer phase})=(W_{1}-W_{2}+W_{S})/\rho_{e}$$

(4) $$ɛ=V_{p}/(V_{p}+V_{s})=(W_{2}-W_{3}-W_{S})/(W_{1}-W_{3})$$

*W*_1_ is the weight of the cylinder filled with ethanol before the immersion of the microsphere sample, *W*_2_ is the weight of the cylinder, the ethanol and the sample after removing the excess ethanol above the 5 mL mark, *W*_3_ is the weight of the cylinder and ethanol after removing the microsphere sample saturated with ethanol, and *W_s_* is the weight of the microsphere sample used in the measurement.

#### 2.6.4. Determination of Residual PVA Content

The % residual PVA content present on the surface of the microspheres prepared using different conditions were determined by the formation of a coloured complex between two adjacent hydroxyl groups of PVA and an iodine molecule [14]. Briefly, 2 mg of the lyophilized microsphere samples prepared using the different conditions were treated with 2 mL of 0.5 M sodium hydroxide (NaOH) for 15 min at 60 °C. Each of the samples was then neutralized with 900 μL of 1 N hydrochloric acid (HCl) and the volume was adjusted to 5 mL with distilled water. To each of the samples, 3 mL of 0.65 M solution of boric acid, 0.5 mL of iodine solution (I_2_)/Potassium iodide (KI) (0.05 M/0.15 M) and 1.5 mL of distilled water were added. Finally, the absorbance of the samples was measured at 690 nm (Novaspec II Visible spectrophotometer, UK) after 15 min of incubation. A standard graph for quantification of PVA was also prepared under identical conditions [14].

#### 2.6.5. Determination of Surface Hydrophobicity

1 mg microsphere samples prepared using different conditions, as mentioned in Section 2.1, were incubated with different concentrations of Rose Bengal dye (4–20 μg/mL) for 3 h at room temperature. The samples were then centrifuged at 110,000 g for 30 min in a microcentrifuge (Sorvall legend RT, UK) to spin down the particles. The supernatant from each of the samples was analysed at 542.7 nm (Novaspec II Visible spectrophotometer, UK) to determine the unbound dye. The dye solution without any microspheres was used as a control and run each time under the same condition to account for the dye bound to the centrifuge tubes [14].

#### 2.6.6. Bovine Serum Albumin (BSA) Adsorption Test

In order to determine the protein adsorption onto the surface of the microspheres 25 mg of the microspheres prepared using different conditions, as mentioned in Section 2.2, were added to 5 mL of distilled water containing 1 mg of BSA. The microspheres were then removed by centrifugation and the concentration of BSA in the supernatant after adsorption on the surface of the microspheres was determined using UV spectroscopy (Eppendorf Biophotometer, UK). The absorbance was measured at 280 nm wavelength. A calibration curve was prepared using known concentrations of BSA. The protein adsorbed (*q*) on the surface of the microspheres was calculated using Equation 3.

(5) $$q=V(C_{i}-C_{f})/m$$

where *C_i_* and *C_f_* are the initial and final BSA concentrations concentration in the supernatant after adsorption studies, respectively; *V* is the total volume of the solution (5 mL); and *m* is the weight of the microspheres added into the solution [15].

#### 2.6.7. Specific Surface Area Measurement

The specific surface area (SSA) of the microspheres was measured according to Brunauer-Emmett-Teller (BET) method, with nitrogen adsorption at 77 K. The physical adsorption of nitrogen gas molecules on a solid surface was measured using a Micromeritics Tristar instrument after degassing for 1 h at 150 °C. The particle diameter (d_BET_) for the P(3HB) microspheres was calculated from the SSA value, using Equation 6:

(6) $$d_{\text{BET}}=6/(\rho \times \text{SSA})$$

where ρ = density of P(3HB), *i.e.*, 1.26 g/cm^3^ and SSA = specific surface area.

#### 2.6.8. Zeta-Potential Analysis

2 g of the P(3HB) microspheres mixed with water were stirred using a magnetic stirrer for 30 min. Following that, the microspheres were centrifuged and re-dispersed in 5 mL of water and stirred once again for 30 min. Water was used as the dispersant for the microspheres to measure their zeta-potential. The zeta-potential analysis of the microspheres was measured using an Agilent 7020 Zeta Probe (Foster City, USA). 1N NaOH and 1N HCl solutions were added as necessary to adjust the pH [15].

#### 2.6.9. X-ray Photoelectron Spectroscopy (XPS)

The surface chemistry of the gentamicin loaded microspheres was analysed using XPS (Cardiff Catalysis Institute, Cardiff). An Axis Ultra DLD system (Kratos Axis Ultra., Cardiff, UK) with the following acquisition parameters; monochromated 120 W X-ray Power (10 mA emission × 12 kV), 20 eV Pass energy, Analysis Area (700 × 300 μm^2^) was used to analyse the samples.

## 3. Results and Discussion

### 3.1. P(3HB) Microsphere Preparation and Characterization

P(3HB) microspheres were prepared by the solid-oil-in-water (s/o/w) technique using different conditions presented in Table 1. In order to optimise the microspheres for maximum encapsulation efficiency the conditions for microsphere preparation including polymer concentration, surfactant concentration and stirring rate were varied. The selected processing conditions, shown in Table 1, were determined using a comprehensive investigation described elsewhere [16].

**Figure 1:**
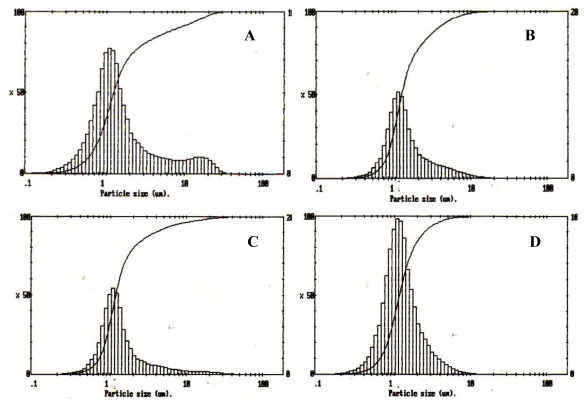
Size distribution analysis of P(3HB) microspheres prepared using different conditions (Table 1). (**A**) sample 1; (**B**) sample 2; (**C**) sample 3; (**D**) sample 4.

The particle size distribution curve of the P(3HB) microspheres prepared using different conditions shown in Table 1 are represented in Figure 1 (A, B, C, D). It was found that the size range of the measured particles was around 1–10 μm, where 90% of the measured particles had a mean particle size ranging from 1.5 to 2.0 μm and 80% of the microspheres had diameters below 5 μm.

### 3.2. Effect of Polymer Concentration on Particle Size

The amount of P(3HB) used per unit volume of the solvent in this study was varied from 1 g to 3 g, respectively. Microspheres prepared under conditions 1 and 2 had an average size of 1.7 μm and 2 μm, as shown in Figure 1A,B respectively. These microspheres were slightly larger than microspheres prepared under conditions 3 and 4 (Figure 1C,D) which were of mean size 1.58 μm and 1.54 μm, respectively. The effect of varying the polymer concentration on the final microsphere structure was studied. The size of microspheres prepared using lower concentrations of P(3HB), such as the microspheres prepared using conditions 1, were slightly smaller than the microspheres prepared using higher concentrations of P(3HB) (condition 2). It was observed that at higher concentrations of the polymer in the emulsion phase, the viscosity of the solution increases. At higher viscosities the solution is difficult to disperse due to which slightly larger microspheres are produced [6,17]. However, microspheres prepared using conditions 4 with polymer concentrations of 3 g/L exhibited a smaller particle size when compared to the microspheres prepared using conditions 3. Although the increasing polymer concentration of 3 g/L in condition 4 had increased the viscosity of the emulsion phase, other factors such as surfactant concentration and stirrer speed would have dominated, contributing to the small size of the microspheres.

### 3.3. Effect of the Surfactant Concentration on Microsphere Size

**Figure 2:**
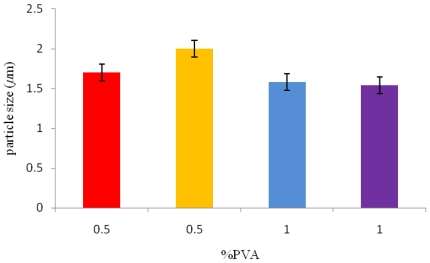
The effect of surfactant concentration on the P(3HB) microsphere sizes.

Polyvinyl alcohol (PVA) concentration, which was used as a surfactant, was varied from 0.5% w/v to 1% w/v, as shown in Table 1. For conditions 1 and 2 (sample 1 and 2, Table 1), a lower PVA concentration of 0.5% w/v was used and in conditions 3 and 4 (sample 3 and 4, Table 1), a PVA concentration of 1% w/v was used in the emulsion phase. The mean particle size, as seen from Figure 2, increased with the decrease in PVA concentration in the emulsion phase.

In addition, the microspheres prepared using conditions 3 and 4 had a smoother finish to the surface and were not coalesced together. An optimal viscosity of the emulsion phase is often required as this prevents the solids from precipitating out of the solution leading to the formation of smoother finished microspheres [6]. For example, in a study conducted by Yang *et al.* [6] it was observed that at lower PVA concentrations such as 0.025% w/v, the water droplets inside the microspheres coalesced with each other, thereby forming interconnecting water channels which increased the release rate of BSA. These water channels then increased in size, eventually leading to the collapse of the microspheres [6]. Higher concentrations of PVA on the other hand are known to increase the viscosity of the external phase, thus making it increasingly difficult to break the emulsion droplets into smaller sizes. Hence, larger microspheres are produced. However, in this study only slight differences in the microsphere sizes were observed with increasing PVA concentrations as there is a maximum PVA concentration which influences the size of the microsphere beyond which an increase in the viscosity (due to the PVA concentration) does not have any further influence on the size of the microspheres and the stirrer speed was found to be the dominant factor.

Once the microspheres were prepared they were washed 3 times with water to remove any residual PVA present on the surface. Residual PVA on the surface of microspheres can have a significant effect on the physical properties, such as surface charge, surface hydrophobicity*, in vitro* drug release rate and rate of protein adsorption [6]. In this study, despite the washings, residual PVA was still found associated with the microspheres. Samples 1 and 2 (conditions 1 and 2) exhibited lower percentages of residual PVA, which were 0.14% and 0.13% when compared to 0.6% and 0.54% of residual PVA present in samples 3 and 4 (conditions 3 and 4, Table 1). After solvent evaporation, PVA is generally known to be bound irreversibly on the surface of the microspheres. The amount of PVA that binds to the curvature of the microspheres is dependent on microsphere size [18]. It was observed by Lee *et al.* [18] that with the decrease in microsphere size and increase in PVA concentration, the PVA transfer rate from the oil/water interface onto the newly formed curved interface of the microsphere increased. This observation was in agreement with the results obtained in this study, where an increase in the PVA content on the surface of the microspheres was observed when the PVA concentration in the external phase was increased. Lee *et al.* [18] also observed that when PVA concentrations of 0.1% w/w, 1% w/w and 10% w/w were used, the PVA content on the surface of the PLGA microspheres was 7.4 mg/g, 15.2 mg/g, and 23.7 mg/g respectively.

### 3.4. Effect of Stirring Rate on Microsphere Size

In order to study the effect of stirring rate on microsphere size, the stirring rate was maintained at 300 rpm and 800 rpm in the final emulsification step under varying PVA and P(3HB) concentrations. The microsphere sizes were 1.7 μm and 1.54 μm when prepared under conditions 1 and 4 (Table 1), with a stirrer speed of 800 rpm. Similarly, the microspheres prepared under conditions 2 and 3 (Table 1), with a stirrer speed of 300 rpm exhibited average sizes of 2 μm and 1.58 μm, respectively. It has been previously observed that at an increased stirring rate of 800 rpm, the microsphere size is significantly reduced [16,19]. From the results obtained in this study, it can be concluded that the stirrer speed may have contributed to the small size of the microspheres. A highly viscous solution is difficult to disperse at a lower stirrer speed, due to which larger sized microspheres are produced. Martin *et al.* [20] observed the formation of larger P(3HB) microspheres of (100 μm to 250 μm) when the stirrer speed was reduced to 500 rpm when compared to the smaller microspheres (5 to 10 μm) produced when the stirrer speed was 800 rpm. Similar observations were made in this study; using a higher stirrer speed of 800 rpm, smaller sized microspheres were produced when compared to those produced at 300 rpm. The stirrer speed should provide the required energy to disperse the oil phase in water producing smaller sized microspheres. It was also observed that the average microsphere size in this study varied from 1 to 5 μm despite the increasing viscosity of the emulsion phase in the presence of both PVA and polymer. In this study, therefore the stirrer speed had a dominant effect on the microsphere size as there is a maximum viscosity which influences the size of the microsphere beyond which an increase in the viscosity due to the PVA and polymer concentration does not have any further influence on the size of the microspheres.

### 3.5. Effect of Stirring on Microsphere Surface Area

**Figure 3:**
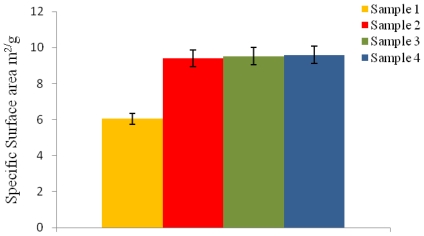
The specific surface area of P(3HB) microspheres prepared using different conditions (Table 1) measured by BET analysis.

The specific surface area of the microspheres varied depending on the different conditions used as represented in Figure 3. An increase in the specific surface area would be expected with a decrease in the average particle size. Microspheres with an average particle size of 1.54 μm and 1.58 μm in diameter (sample 3 and sample 4, Table 1) exhibited a specific surface area of 9.60 m^2^/g and 9.52 m^2^/g, respectively. Whereas, microspheres with slightly larger particle size of 2 μm and 1.7 μm in diameter (sample 1 and sample 2, Table 1), exhibited a decrease in the specific surface area being 6.05 m^2^/g and 9.4 m^2^/g.

Microspheres with a higher specific surface area also exhibited an increase in the higher % residual PVA adsorbed on the surface. Researchers like Poletto *et al.* [21] have also observed an increase in the specific surface area, from 33 m^2^/g to 120 m^2^/g, of P(3HB-co-3HV) microspheres when the particle size was reduced from 360 μm to 190 μm.

### 3.6. Surface Morphology

**Figure 4:**
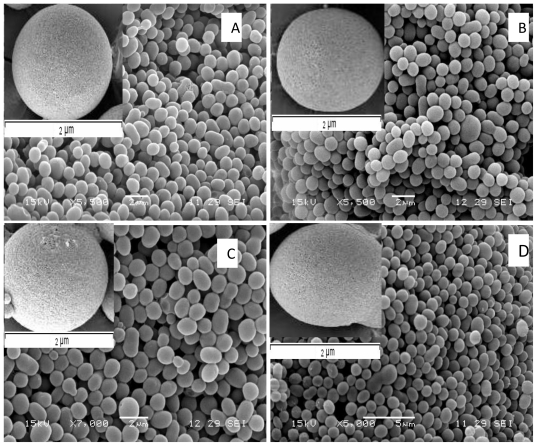
(SEM) of P(3HB) microspheres prepared under different conditions described in Table 1: (**A**) sample 1; (**B**) sample 2; (**C**) sample 3; (**D**) sample 4.

The surface morphology of microspheres using different conditions was observed by SEM (Figure 4). Microspheres prepared under the different conditions (Table 1), appeared spherical in shape, with a smooth surface morphology.

The type of solvent is known to influence the surface morphology of microspheres. In this study chloroform was chosen as the solvent as opposed to dichloromethane due to lesser miscibility of chloroform in water, which is 1 in 200 parts when compared to dichloromethane, which is 1 in 50 parts [14]. An increased amount of PVA is known to partition into the polymer phase when the organic solvent exhibits an increased miscibility in water because of higher hydrophilicity [14]. Thus in order to avoid a higher deposition of residual PVA on the surface of the microspheres chloroform was used as the solvent.

### 3.7. Surface Hydrophobicity

**Figure 5:**
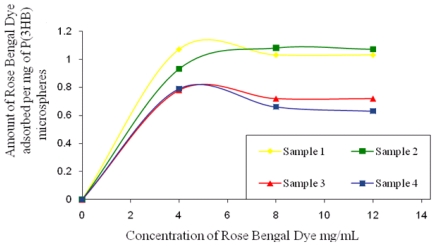
Amount of Rose Bengal Dye bound onto the surface of P(3HB) microspheres (sample1, sample 2, sample 3, sample 4) as a function of an increase in Rose Bengal dye concentration.

The surface hydrophobicity of microspheres fabricated using higher and lower concentrations of PVA was determined by comparing the amount of hydrophobic Rose Bengal dye that was adsorbed per mg of the microspheres. As seen in Figure 5, the amount of Rose Bengal bound to the surface of the microspheres decreases with the increase in PVA concentration. Microspheres fabricated under conditions 3 and 4 (Table 1) exhibited a higher % residual PVA and hence bound a lower amount of Rose Bengal dye when compared to the microspheres fabricated under conditions 1 and 2 (Table 1). For example, at the highest concentration of 12 mg/mL of Rose Bengal dye, the microspheres prepared under condition 3 and 4 bound 0.63 mg/mg and 0.72 mg/mg of the dye in contrast to the higher values of 1.03 mg/mg and 1.07 mg/mg measured for microspheres prepared under conditions 1 and 2.

Microspheres fabricated with a higher PVA (1%) concentration appeared to be more hydrophilic in nature as compared to those that were formulated with 0.5% PVA, as seen from the lesser amounts of Rose Bengal Dye bound to their surfaces. In a relevant study Sahoo *et al.* [14] observed a lower binding of 0.005 μg/mL of Rose Bengal dye on the surface of nanoparticles which were formulated with 5% PVA when compared to 0.024 μg/mL bound on nanoparticles formulated with a 0.5% PVA concentration. During microsphere preparation, hydrophobic ends of PVA penetrate into the organic phase and interact with P(3HB) microspheres due to which an irreversible binding of PVA occurs on the microsphere surface when the solvent evaporates. An increased amount of PVA is known to partition into the polymer phase when the organic solvent exhibits an increased miscibility in water because of higher hydrophilicity. Since PVA is a hydrophilic polymer, the higher amount of residual PVA on the surface of the microspheres would have accounted for the higher hydrophilicity.

### 3.8. Zeta-Potential Analysis

**Figure 6:**
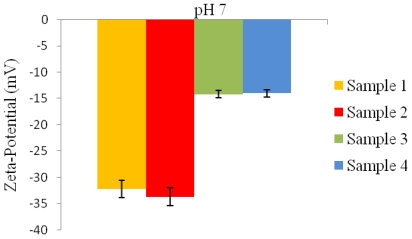
Zeta-potential values of P(3HB) microspheres fabricated using different conditions (Table 1) at pH 7.

Microspheres prepared with a higher PVA concentration in conditions 3 and 4 (Table 1), at pH 7 exhibited much lower negative zeta-potential values of −14.2 mV and −14 mV when compared to the zeta-potential values of −32.2 mV and −33.7 mV for the microspheres prepared under conditions 1 and 2 (Table 1), as seen in Figure 6.

**Figure 7:**
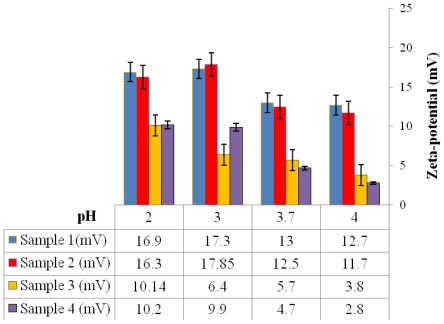
Zeta-potential values of P(3HB) microspheres fabricated using different conditions (Table 1) at different pH values.

From the zeta-potential-pH profiles it was observed that all microspheres exhibited a negative surface charge at pH 7, as shown in Figure 6. However at a lower pH, the microspheres exhibited a positive zeta-potential value (Figure 7). Microspheres fabricated using conditions 3 and 4 (Table 1) exhibited lower positive zeta-potential charge when compared to microspheres fabricated using conditions 1 and 2 (Table 1).

The zeta-potential values reflect the change in the surface charge due to the deposition of PVA on the surface of the microspheres [22]. The COOH groups on microsphere surfaces exhibit a negative charge due to deprotonation at pH 7; however at a lower pH a charge reversal from negative to positive was observed. This phenomenon was observed for all microspheres prepared using different conditions. Similarly, decrease in positive zeta-potential values were observed when the concentration of residual PVA on the surface of microspheres was increased. Often non-ionic surfactants such as PVA are known to strongly adhere on the microsphere surface by anchoring the hydrophobic tail into the polymer only when it is hydrophobic, leaving the polar head protruded on the surface [14]. During microsphere preparation, hydrophobic ends penetrate into the organic phase and interact with P(3HB) microspheres due to which an irreversible binding of the PVA occurs on the surface of microspheres when the solvent evaporates. Thus, when P(3HB) is fabricated into microspheres, PVA used as a surfactant coats the surface thereby shielding the surface charge of PHB. Due to this effect, P(3HB) processed into microspheres carries a much less negative charge at neutral pH when compared to the negative charge of the neat P(3HB) (−40 mV at neutral pH). In acidic solutions such as pH < 4.0, C=O of the ester present on the surface of microspheres gets protonated. As a result, at lower pH the negative surface charge is reversed to a positive surface charge. Similar observations were made by Lee *et al.* [18] who showed that with an increase in PVA concentration from 0.5% to 5%, the zeta-potential values of the PLGA nanoparticles decreased from −15.4 mV to −8.0 mV at neutral pH. However in acidic solutions (pH < 5.0), the zeta-potential values of microspheres with lower PVA concentration (0.5% w/v) showed a relatively high positive charge when compared to the lower surface charge of microspheres prepared with higher PVA concentration (1% w/v) [18].

### 3.9. Effect of Zeta-Potential on Protein Adsorption

**Figure 8:**
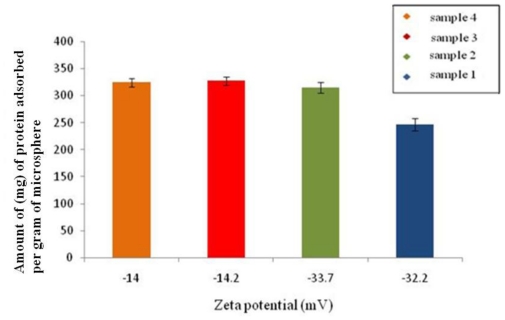
Amount of BSA adsorbed on the surface of P(3HB) microspheres (sample 1, sample 2, sample 3, sample 4) as a function of zeta-potential.

The changes in the surface properties of the microspheres can have a significant impact on their use in biological applications [15]. Thus in this study protein adsorption in the presence of varying surface charges was studied. The comparison of protein (BSA) adsorption and zeta-potential of microspheres fabricated under different conditions is represented in Figure 8. It is observed that, microspheres prepared under conditions 3 and 4 (Table 1), adsorbed a slightly higher amount of protein, *i.e.*, 327 mg/g and 324.4 mg/g, when compared to 315 mg/g and 240 mg/g, the amount adsorbed on the microspheres prepared under conditions 1 and 2.

In this study, a higher amount of protein was adsorbed on microspheres with a lesser negative charge when compared to those with a higher negative charge. BSA adsorption on the surface of an adsorbent at neutral pH occurs due to electrostatic interaction. Since BSA is negatively charged at neutral pH and the microspheres fabricated using different conditions also exhibited a negative surface charge at neutral pH, other interactive factors such as van der Waals forces, and steric interactions would have contributed to the protein adsorption [23]. Similar observations were made by Patil *et al* [15] where 10 mg/mg (nanoparticles) of BSA adsorption on nanoparticles with a negative charge of −17.72 mV was observed. However, 67.40 mg/mg of BSA was adsorbed on cerium oxide nanoparticles with a zeta-potential of 59.32 mV. The authors observed an increasing BSA adsorption on nanoparticles with a positive zeta-potential charge, confirming that the electrostatic interaction was the primary reason for protein adsorption [15].

### 3.10. Determination of the Drug Encapsulation Efficiency in P(3HB) Microspheres

In order to achieve higher drug loadings, the effects of polymer concentration, PVA concentration and stirring rate on encapsulation efficiency during microsphere preparation was investigated. In this study, gentamicin was investigated with an initial drug loading of 2 mg/g in P(3HB) microspheres fabricated using the different conditions described in section 2.1.

Microspheres prepared under condition 4, with the smallest particle size, exhibited the highest encapsulation efficiency of 48% when compared to microspheres produced using other conditions. Sendil *et al.* [24] also observed an increase in the encapsulation efficiency with a decrease in microsphere size. P(3HB-co-3HV) microspheres with an average diameter of 399 μm exhibited lower encapsulation efficiency of (20.2%) when compared to 30.1% encapsulation efficiency exhibited by smaller microspheres (340 μm) [24]. It was suggested that the faster rate of solvent removal in microspheres with a smaller size led to the rapid entrapment of the drug. Similarly, in this study the highest entrapment of a highly hydrophilic drug such as gentamicin occurred in the microspheres with the smallest particle size due to the rapid solvent evaporation rate.

Microspheres fabricated with higher concentrations of PVA are known to yield stable emulsions which prevent the transfer of the encapsulated hydrophilic drugs/proteins into the surroundings; as a result the drug/protein is more evenly distributed within the interior of the microspheres [6]. This could also explain the higher encapsulation efficiency of gentamicin in P(3HB) microspheres fabricated with a higher PVA concentration used in this study, when compared to the lower encapsulation of 11% observed within P(3HB) microspheres fabricated with a lower PVA concentration.

Also, variations in the polymer/drug ratio influence the encapsulation efficiency [22]. In the presence of high concentrations of the polymer and fixed drug concentrations, higher concentrations of the drug are encapsulated within the microspheres [22]. This could be another reason why higher encapsulation efficiency was observed in sample 4 (Table 1), where an increased polymer concentration of 3 g was used. Microspheres prepared by condition 4, Table 1, exhibiting the highest encapsulation efficiency, were chosen for further drug release studies.

### 3.11. Determination of Drug-Polymer Interaction

**Figure 9:**
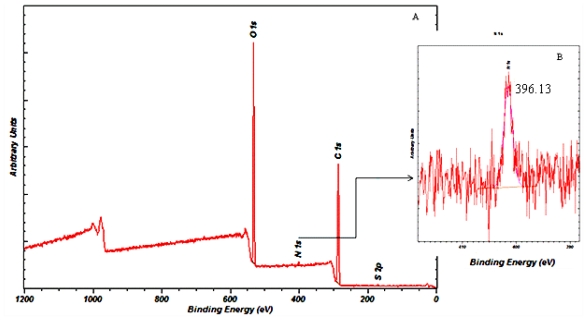
(**A**) Typical XPS spectra of gentamicin loaded P(3HB) microspheres; (**B**) Magnification of the nitrogen peak with a binding energy of 396.13 eV.

The surface chemistry of microspheres with the highest encapsulation efficiency of 48% was investigated using XPS. As shown in Figure 9, the presence of carbon, nitrogen and oxygen on the surface of the gentamicin-loaded microspheres was examined using soft X-Ray excitation. Nitrogen present in gentamicin was used to estimate the amount of drug entrapped during the microsphere fabrication procedure. The surface chemical composition of the tested sample was found to be: 67.29% carbon, 31.25% oxygen and 1.05% nitrogen. The binding energy of nitrogen N-1s is *E_b_* = 399.5 eV which corresponds to the single bonded nitrogen, as shown in Figure 9B. The binding energy of nitrogen N-1s present on the gentamicin-loaded microspheres was found to be 396.13 eV, which was quite close to the value for singly bonded nitrogen.

The assessment of gentamicin adsorption on the microsphere surface prepared under condition 4 (Table 1) was done on the basis of nitrogen content present on the samples since nitrogen is unique to the drug and is absent in the P(3HB) microspheres. From the XPS data a significant increase in nitrogen content on the surface of gentamicin loaded samples, was observed, when compared to unloaded P(3HB) microspheres, where, as expected, only carbon and oxygen were detected. This result therefore indicated that apart from being encapsulated, some amount of gentamicin was bound onto the microsphere surface. Similar results were observed by Naraharisetti *et al.* [9] who also observed the presence of surface bound drugs (gentamicin) on microspheres made using poly-l-lactide (PLLA) and dl-lactic-co-glycolic acid (PLGA).

### 3.12. *In Vitro* Release Studies

**Figure 10:**
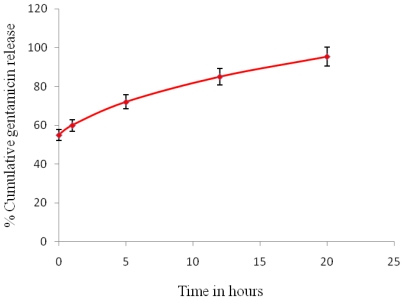
*In vitro* gentamicin release profile from P(3HB) microspheres in SBF (sample 4).

The cumulative *in vitro* release of gentamicin from P(3HB) microspheres prepared using condition 4 (sample 4) is shown in Figure 10. In this study the release profile is seen to be bimodal, with an initial burst release followed by controlled continuous release. Gentamicin released from microspheres in the SBF solution at 37 °C and pH 7.4 was measured after periods of 1 h, 5 h, 12 h and 20 h. During the initial burst release phase (0 min to 1 h) the amount drug released was 90.6 μg/mL, which was 60% of the total encapsulated drug. This phase was followed by the controlled release period which occurred from 5 h to 12 h, where small amounts of the drug, *i.e.*, 24.6 μg/mL to 18 μg/mL, slowly diffused from the microspheres into the release buffer. Finally, after 20 h, the cumulative drug release was 95.33%.

The *in vitro* drug release rate is largely dependent on the size of the microspheres [6]. Larger microspheres (100–300 μm) tend to release the drug slowly and for a much longer time when compared to smaller microspheres [25]. This slow release is due to the reduced drug diffusion path within the microspheres and the decreased specific surface area of larger microspheres when compared to smaller microspheres. Thus in this study, drug release within a period of 24 h was observed which can probably be attributed to the small size (1.54 μm in diameter) of the microspheres.

The *in vitro* drug release is also dependent on the drug distribution within the microspheres. The high initial burst release is often attributed to the presence of surface associated drugs [17]. In this study gentamicin, in addition to being encapsulated within the microspheres, was also adsorbed on the surface of the microspheres, as confirmed by XPS analysis. As a result, an initial burst release of 60% of the total encapsulated drug was recorded. After the initial burst release, a slow and controlled release of the drug was observed. Often factors such as the type of encapsulated drug and polymer determine the rate of slow and sustained release [21]. In this study, a sustained release phase from 5 h to 12 h was observed where small amounts of the drug slowly diffused from the microspheres into the release buffer. The length of the sustained release phase of polymeric matrices is largely dependent on the type of polymer used [9]. Polymers are known to undergo degradation either by surface erosion or bulk degradation or a combination of both. With the increase in the penetration of water molecules into the polymeric matrix, homogenous surface erosion starts to occur which is followed by bulk degradation. In this study, the total drug cumulative release of 95.33% was found to occur within a period of 20 h, therefore, drug release by either surface erosion or degradation was not considered due to the slow degradable nature of the polymer. The drug release in the final stage occurred most likely through the water channels that were created by the diffused drugs. Further in this study it was observed that in the presence of the hydrophilic drug and PVA, the water uptake rate of the polymer was accelerated, thereby having a significant impact on drug release.

## 4. Conclusions

A solid-in-oil-water (s/o/w) technique was used to produce tailored poly(3-hydroxybutyrate) P(3HB) microspheres. The effect of several parameters such as polymer concentration, surfactant concentration and stirring rate were investigated regarding their influence on microsphere properties, shape and dimensions. The average size of the microspheres varied from 2 μm to 1.54 μm with specific surface areas from 9.60 m^2^/g to 6.05 m^2^/g. Microspheres produced using low stirring rates of 300 rpm exhibited slightly larger size than the microspheres produced when the stirring rate was increased to 800 rpm. The presence of residual PVA on the surface of the microspheres affected several properties of microspheres such as zeta-potential, surface hydrophobicity, protein adsorption and encapsulation efficiency. The surface and internal morphology, drug distribution and release kinetics of microspheres exhibiting the highest encapsulation efficiency (48%), were investigated. The hydrophilic nature of the drug also had an influence on the drug release behaviour. The *in vitro* release of gentamicin in SBF was bimodal where an initial burst release was observed followed by a diffusion mediated sustained release. The knowledge gained in this work about the different factors influencing the drug release from P(3HB) microspheres will have important implications for the development of drug delivery vehicles exhibiting controlled delivery of potent drugs for various medical applications and for tissue engineering therapeutics.

## Figures and Tables

**Table 1:** The varying processing conditions (amount of surfactant, polymer concentration and stirring rate) used for the synthesis of P(3HB) microspheres.

Sample	Polymer Concentration (g/L)	PVA Concentration (%)	Stirring Rate (rpm)
1	1	0.5%	800
2	3	0.5%	300
3	1	1%	300
4	3	1%	800
